# An Intranasal Challenge Model in African Green Monkeys (*Chlorocebus aethiops*) for Mild-to-Moderate COVID-19 Disease Caused by Subvariant XBB.1.5

**DOI:** 10.3390/v17101373

**Published:** 2025-10-14

**Authors:** Nadia Storm, Ming Lo, Nicholas Crossland, Margaux Seyler-Schmidt, Hilary Staples, Daniela Silva-Ayala, Ambre M. Laprise, Lauren St. Denis, Kyle Grosz, Aoife O’Connell, Hans Gertje, Tillie Ripin, Claire Decker, M. Mazur, Colleen Thurman, Marlene Espinoza, Gavin Morrow, Christopher L. Parks, Christopher L. Cooper, Anthony Griffiths

**Affiliations:** 1 National Emerging Infectious Diseases Laboratories, Boston University Chobanian and Avedisian School of Medicine, Boston, MA 02118, USA; nstorm@bu.edu (N.S.); minglo@bu.edu (M.L.); ncrossla@bu.edu (N.C.); margaux@bu.edu (M.S.-S.); hstaples@bu.edu (H.S.); d.silvaayala@missouri.edu (D.S.-A.); alaprise@bu.edu (A.M.L.); lstdenis@bu.edu (L.S.D.); kgrosz@bu.edu (K.G.); aocon@bu.edu (A.O.); hgertje@bu.edu (H.G.); tripin@bu.edu (T.R.); cmldeck@gmail.com (C.D.); mmazur@bu.edu (M.M.); cthurman@bu.edu (C.T.); 2 Department of Virology, Immunology and Microbiology, Boston University Chobanian and Avedisian School of Medicine, Boston, MA 02118, USA; 3 Department of Pathology and Laboratory Medicine, Boston University Chobanian and Avedisian School of Medicine, Boston, MA 02118, USA; 4 Department of Molecular Microbiology and Immunology, Bond Life Sciences Center, and Laboratory for Infectious Disease Research, University of Missouri School of Medicine, Columbia, MO 65211, USA; 5 International AIDS Vaccine Initiative, New York, NY 10004, USA; mespinoza@iavi.org (M.E.); gavin.morrow@gmail.com (G.M.); cparks@iavi.org (C.L.P.); christopher.cooper@tonixpharma.com (C.L.C.); 6 Tonix Pharmaceuticals, Frederick, MD 21701, USA

**Keywords:** SARS-CoV-2, African green monkey, animal model, intranasal exposure, Omicron XBB.1.5

## Abstract

Severe acute respiratory syndrome coronavirus 2 (SARS-CoV-2) primarily causes mild to moderate respiratory illness in humans, but infection can also lead to long-term complications, including chronic fatigue, respiratory and cardiac issues, or even death. In November 2021, the emergence of the highly transmissible Omicron variant marked a significant shift in the pandemic, with its subvariants rapidly spreading and continuing to evolve worldwide. The continuing introduction of Omicron subvariants underscores the need for the development of up-to-date vaccines, as well as for appropriate animal models in which they can be evaluated. Among these subvariants, XBB.1.5 stands out for its ability to evade the immune response from previous infection or vaccination. The objective of this study was to determine the disease course in African green monkeys (AGMs) following intranasal exposure to the XBB.1.5 subvariant. In four intranasally exposed AGMs, histopathological findings in the lungs consistent with SARS-CoV-2 infection included lymphohistiocytic and neutrophilic bronchiolitis with variable numbers of syncytial cells, to terminal bronchiole-centric, bronchointerstitial pneumonia with alveolar type II (AT2) pneumocyte hyperplasia, with evidence of acute alveolar injury, including alveolar septal necrosis and hyaline membrane formation. The two males showed more severe pneumonia compared to the two females. SARS-CoV-2 RNA was detected in the lungs or tracheobronchial lymph nodes in the males but not in the females, which correlated with higher cumulative lung pathology scores in the males. In the females, SARS-CoV-2 RNA was limited to the colon and nasal turbinates. Our results indicate that AGMs exhibit a disease course similar to most humans when exposed intranasally, making them a suitable model for studying mild to moderate SARS-CoV-2 infection. Therefore, further work is warranted to determine if this model could have utility for the evaluation of vaccine and therapeutic candidates against contemporary SARS-CoV-2 variants.

## 1. Introduction

The virus responsible for coronavirus disease 2019 (COVID-19), known as severe acute respiratory syndrome coronavirus 2 (SARS-CoV-2), is an enveloped, positive-sense, single-stranded RNA virus. It primarily spreads through respiratory droplets [1] and can cause symptoms ranging from mild, flu-like conditions to severe respiratory distress [2]. The virus emerged towards the end of 2019 [3] and within months became a global pandemic, causing widespread illness, severe economic losses, and millions of deaths [4]. Since its emergence, SARS-CoV-2 has rapidly and persistently evolved to produce novel variants with varying degrees of transmissibility, immune evasion and lethality. In November 2021, Omicron was reported for the first time in South Africa as a new variant of concern due to 37 mutations in the spike protein that allowed the variant to spread more easily and evade immunity provided by previous infection or existing vaccine-induced immunity [5]. Now several years later, Omicron subvariants continue to circulate and evolve, posing ongoing challenges in managing COVID-19 infections globally.

Among the SARS-CoV-2 Omicron subvariants, XBB.1.5 garnered attention for its enhanced transmissibility and resistance to existing monoclonal antibody therapies [6,7], prompting ongoing monitoring and research to understand its impact on public health and to adjust treatments, vaccines and vaccination strategies as necessary. To understand the host immune response and the pathogenesis of disease caused by SARS-CoV-2 Omicron subvariants, and to develop and evaluate new medical countermeasures against infection, animal models are required that accurately recapitulate human disease. Currently available data suggest that disease in experimentally infected African green monkeys (AGM) mimics the mild disease course seen in those of young and healthy humans, with animals developing a low-grade fever, lung inflammation, and viral shedding from mucous membranes and the gastrointestinal tract [8,9]. While both rhesus macaques and AGMs are susceptible to SARS-CoV-2, despite differences at one key residue in the SARS-CoV-2 receptor ACE2 (E31 versus K31) in human and rhesus macaques [10], AGMs have been shown to have an interferon gene signature that is more similar to the antiviral responses seen in SARS-CoV-2-infected humans [11]. Further, in rare instances, a more severe acute respiratory distress syndrome (ARDS) has been reported in AGMs, indicating that this model more accurately replicates severe COVID-19 disease sometimes seen in humans [12]. However, evaluation of more recent subvariants of the Omicron lineage have not yet been reported in the literature for this model. In addition to these biological and clinical considerations, practical factors support the use of AGMs. Global demand for rhesus and cynomolgus macaques has limited their availability, whereas AGMs remain comparatively more accessible, making them a feasible alternative nonhuman primate model.

In this study, four AGMs were exposed by the intranasal (IN) route to a target dose of 1 × 10^5^ plaque forming units (PFU) of the Omicron subvariant XBB.1.5 of SARS-CoV-2. Animals were monitored for disease progression up to 5 days post-infection (dpi). This early termination timepoint was selected to capture acute phase responses and early viral kinetics, whilst targeting maximum pathology during the estimated peak period of viral replication. Plasma, serum, whole blood, mucosal samples (nasal and oral secretions), and bronchioalveolar lavage fluid (BALF) were collected at various timepoints, and tissues were collected at necropsy at the end of the in-life phase of the study (5 dpi). All animals developed pulmonary lesions consistent with SARS-CoV-2 infection, although with variable severity. Fever was only present in a single animal which also presented with more severe gross and microscopic pathology findings. While the study was limited in scope and preliminary in nature, our results indicate susceptibility of AGMs to an Omicron variant of SARS-CoV-2 and broadens the understanding of SARS-CoV-2 pathogenesis in this model of disease. With further study, the IN AGM model could be used to assess host immune responses against SARS-CoV-2, investigate viral pathogenesis, and evaluate novel medical countermeasures against emerging variants of the virus.

## 2. Materials and Methods

### 2.1. Ethics Statement

This study was performed following standard operating procedures approved by the Boston University Institutional Biosafety Committee in the AAALAC-accredited animal biosafety level four (ABSL-4) laboratory at the National Emerging Infectious Diseases Laboratories of Boston University. Experiments were performed following recommendations described in the Guide for the Care and Use of Laboratory Animals [13] and was approved by the Boston University Institutional Animal Care and Use Committee (IACUC, protocol number 202000034). An experimental protocol was submitted to and approved by the U.S. Army Medical Research Acquisition Activity (USAMRAA) Animal Care and Use Review Office (ACURO) prior to the commencement of the experiment.

### 2.2. Animal Care

This study consisted of four Caribbean-origin AGMs (*Chlorocebus aethiops*) (two females, Subjects 01 and 02, and two males, Subjects 03 and 04) of approximately seven years of age and weighing between 3.76 and 6.38 kg on the day of infection. Animals were experimentally naïve and tested negative for tuberculosis, simian immunodeficiency virus (SIV), simian T-lymphotrophic virus-1 (STLV-1), simian retrovirus (SRV1 and SRV2), filoviruses, SARS-CoV-2 and macacine herpes virus 1 (herpes B virus). Prior to infection, animals were evaluated by a veterinarian to confirm health and were transferred to the ABSL-4 facility to acclimate for five days. Animals were single-housed in stainless steel cages conforming to the Guide [13]. Water was available ad libitum, and animals were fed 5048 Certified Primate Diet (LabDiet, Marlborough, MA, USA) twice daily. Food-based enrichment such as fresh fruits and vegetables were provided at least once a day. Cage pans, floors and room floors were cleaned daily. Targeted environmental and photoperiod conditions included a temperature of 25 °C ± 5 °C, relative humidity of 30% to 70%, and a light cycle of 12 h on/12 h off. Animals were observed by veterinary staff at least twice daily, 6 h ± 2 h apart for morbidity and mortality. Disease progression was documented using a clinical score sheet with the following scoring criteria: 0 = alert, responsive, normal activity, free of disease signs or exhibits only resolved/resolving disease signs; 1 = slightly diminished general activity, subdued but responds normally to external stimuli; 2 = withdrawn, may have head down, or fetal posture, or hunched, or reduced response to external stimuli; 3 = recumbent but able to rise if stimulated, or moderate to dramatically reduced to response to external stimuli; 4 = persistently recumbent, or severely or completely unresponsive, or may have signs of severe respiratory distress.

### 2.3. Viruses and Cell Lines

Vero E6 cells (NR-596; BEI Resources, Manassas, VA, USA) used for plaque assays were maintained in Dulbecco’s Modified Eagle Medium (Gibco, Grand Island, NY, USA) supplemented with 2 mM L-glutamine (Gibco, Grand Island, NY, USA), 1 mM of sodium pyruvate (Gibco, Grand Island, NY, USA), and 10% heat-inactivated fetal bovine serum (Gibco, Grand Island, NY, USA) at 37 °C with 5% CO_2_.

The hCoV-19/USA/MD-HP40900/2022 (lineage XBB.1.5; Omicron variant) isolate of SARS-CoV-2 (NR-59105, BEI Resources, Manassas, VA, USA) was used as the exposure virus directly without any further amplification. The virus was isolated from a human on 29 November 2022, in Maryland, USA and subsequently cultured on Vero E6 *Chlorocebus aethiops* kidney epithelial cells modified to over-express transmembrane protease, serine 2 and human angiotensin-converting enzyme 2.

### 2.4. Anesthesia

Animals were sedated prior to virus exposure, all physical examinations, and phlebotomy via intramuscular injection of ketamine (Dechra Pharmaceuticals, Northwich, UK) at a dose of 14 mg/kg.

### 2.5. Virus Exposure and Verification

The SARS-CoV-2 XBB.1.5 virus stock (titer 2.27 × 10^5^ PFU/mL) was diluted to a target concentration of 2 × 10^5^ PFU/mL in sterile Dulbecco’s Phosphate-Buffered Saline (PBS; Gibco, Waltham, MA, USA). Prior to animal infection, an aliquot of the prepared exposure material was removed for verification of titer via an Avicel-based crystal violet plaque assay [14] and the potency was in the expected range based on the titer provided by the supplier. For a target dose of 1 × 10^5^ PFU, a total of 0.5 mL of exposure material was administered to each animal IN using a pipette, delivered as 0.25 mL per naris. Intranasal instillation was chosen as a well-established method for delivering controlled delivery of a defined inoculum. The virus was instilled in rhythm with the animal’s inspiration while keeping the head tilted back to ensure inhalation of the virus. The verified exposure dose titer was determined to be 6.75 × 10^4^ PFU/mL, which was within the expected range of variability for the plaque assay (±0.5 log_10_).

### 2.6. Sample Collection

Rectal temperature, body weight, oral and nasal swabs, whole blood and serum were collected at 0, 1, 3 and 5 dpi. Nasal and throat swabs were collected for viral load analysis by rotating a sterile swab (Becton, Dickinson and Company, Franklin Lakes, NJ, USA) around the inner walls of both nares and the oropharynx, followed by placement of the swab into universal transport medium provided with the kit. Venous blood for hematology was collected into tubes containing ethylenediaminetetraacetic acid (EDTA). Plasma samples for cytokine and chemokine analysis were prepared from whole blood collected in a 3.2–3.8% sodium citrate tube, and whole blood collected into a serum separator tube was processed to obtain serum for assessment of viremia and clinical chemistry.

Bronchoalveolar lavage fluid (BALF) collection is a standard procedure in nonhuman primates, and when performed carefully induces only minimal and localized effects. BALF was collected at 0 and 5 dpi. To collect BALF, a laryngoscope was used to intubate the animal with an endotracheal tube. A catheter was threaded through the lumen of the tube and up to four aliquots of 10 mL of sterile saline instilled into the lower respiratory tract and collected at each sampling event. The animals were vigorously coupaged and the BALF recovered by drawing back on the syringe. The recovered BALF was transferred to a 50 mL conical tube which was centrifuged for 10 min at 1500× *g* at ambient temperature. The supernatant was removed, aliquoted and stored between −60 °C and −90 °C until processing.

### 2.7. RNA Extraction and RT-qPCR

Ribonucleic acid was extracted from serum, swab, BALF, and tissue samples using the Quick-RNA Miniprep kit (Zymo Research, Atlanta, GA, USA) according to the manufacturer’s instructions. Prior to RNA extraction, tissues were trimmed to approximately 100 mg and homogenized in 1 mL Trizol (Invitrogen, Carlsbad, CA, USA) using the QIAGEN TissueLyser II (QIAGEN, Germantown, MD, USA), followed by centrifugation at 5000× *g* for 1 min at ambient temperature to clarify the supernatant. Quantitative reverse transcription polymerase chain reaction (RT-qPCR) was performed as previously described [15].

### 2.8. Crystal Violet Plaque Assay

Monolayers of Vero E6 cells and an Avicel RC591 (DuPont Nutrition and Biosciences, Wilmington, DE, USA) overlay-based crystal violet plaque assay was performed to determine viral burden in serum, BALF and tissue samples as described previously [14], with plates incubated for 3 days. Prior to use in the assay, tissues were trimmed to approximately 100 mg and homogenized in 1 mL Dulbecco’s Modified Eagle Medium (DMEM) (Gibco, Grand Island, NY, USA) supplemented with 2% heat-inactivated fetal bovine serum (FBS) (Gibco, Germantown, MD, USA) using the QIAGEN TissueLyser II (QIAGEN, Germantown, MD, USA).

### 2.9. Cytokine and Chemokine Analysis

Cytokine and chemokine analysis was performed on plasma and BALF samples using the NHP XL Cytokine Luminex^®^ Performance Premixed Kit (12-Plex) with the BioPlex200 (BioRad, Hercules, CA, USA) according to the manufacturer’s instructions. The following cytokines and chemokines were analyzed: monocyte chemoattractant protein-1 (CCL2/JE/MCP-1), C-X-C motif chemokine 10 (CXCL10/IP-10/CRG-2), granulocyte colony-stimulating factor (G-CSF), interferon (IFN) alpha, IFN-beta, IFN-gamma, interleukin (IL)-1 beta/IL-1F2, IL-6, IL-8/CXCL8, IL-12 p70, tumor necrosis factor (TNF)-alpha, and vascular endothelial growth factor (VEGF).

### 2.10. Clinical Pathology

Complete blood counts were performed on EDTA whole blood using the Abaxis VetScan HM5 (Zoetis, Parsippany, NJ, USA). Clinical chemistry analysis was performed on serum using the Abaxis Piccolo Biochemistry Panel Plus rotor with the Piccolo Xpress Analyzer (Zoetis, Parsippany, NJ, USA). Clinical chemistry markers analyzed included C reactive protein, glucose, blood urea nitrogen, creatinine, uric acid, calcium, albumin, total protein, alanine aminotransferase, aspartate aminotransferase, alkaline phosphatase, gamma glutamyltransferase, and amylase.

### 2.11. Histopathology

All animals were humanely euthanized at the end of the study (5 dpi). At necropsy, a gross pathology examination of external surfaces and orifices, cranial, thoracic, and abdominal cavities and all organs therein was performed. The following tissues were aseptically collected for either viral load or histopathologic analysis: individual lung lobes including the right cranial, middle, caudal, and accessory lung lobes, and left cranial, and left caudal lung lobes; tracheobronchial lymph node, nasal turbinates, tonsil, brain including the frontal lobe, cerebellum, brainstem and olfactory bulb, heart, spleen, liver, kidney, axillary lymph node, inguinal lymph node, submandibular lymph node, colon, and any gross lesions. Samples for histopathology were fixed in 10% neutral-buffered formalin for 72 h prior to being removed from biocontainment, processed using an automated Tissue Tek VIP 5 vacuum infiltration processor (Sakura, Torrance, CA, USA), and embedded as formalin fixed paraffin embedded (FFPE) blocks using a HistoCore Acadia paraffin embedding station (Leica, Wetzlar, Germany). From each FFPE block, a 5-μm tissue section was generated using a RM2255 automated microtome (Leica, Wetzlar, Germany) and stained with hematoxylin and eosin using a ST5020 autostainer (Leica, Wetzlar, Germany). Slides were examined microscopically by a board-certified veterinary pathologist (ML), and peer-reviewed by another board-certified veterinary pathologist (NC). A summary of the lung ordinal scoring criteria is provided in Table S3. In all subjects, there was concurrent moderate eosinophilic bronchitis targeting larger conducting airways of variable severity, which was interpreted as a separate, likely hypersensitivity response of unknown origin. To avoid the potential contribution of this inflammatory response to the reported lung ordinal scoring, inflammation of larger conducting airways (bronchi) was excluded from the lung ordinal scoring criteria. In addition, due to the potential confounding effect of euthanasia artifacts, pulmonary edema and erythrocyte extravasation were also excluded from the lung ordinal scoring criteria. Individual animal lung ordinal scores of each lobe and their cumulative scores were provided in Table S4. Based on the lung ordinal scoring criteria (Table S3), the theoretical maximal cumulative lung ordinal score of each lung lobe is 24, and the theoretical maximal total lung ordinal score of each animal is 144.

### 2.12. Immunohistochemistry and Digital Quantification of Immunohistochemical Staining

Immunohistochemistry (IHC) was performed on a Ventana Discovery Ultra autostainer (Roche Diagnostics, Indianapolis, IN, USA). Pretreatment was performed with Benchmark Ultra CC2 (950-223, Roche), a citrate-based antigen retrieval buffer, at 91 °C for 32 min in the DAB protocol, and Benchmark Ultra CC1 (950-224), a tris-based antigen retrieval buffer, was used at 95 °C for 32 min in the Pro-SPC/SARS-CoV-1/2 N protein duplex protocol. The primary antibody of SARS-CoV-1/2 N protein (clone E8R5W/1C7C7, Cell Signaling Technology, Danvers, MA, USA) was used at a dilution of 1:1000 in SignalStain^®^ antibody diluent (#8112L, Cell Signaling Technology, Danvers, MA, USA) and incubated at room temperature for 40 min. Signals were developed with chromogen 3,3′-diaminobenzidine (DAB) (760-159, Roche Diagnostics) or Discovery purple (760-229, Roche Diagnostics). The primary antibody of prosurfactant C (ProSP-C) protein (clone WRAB-9337, Seven Hills Bioreagents, Cincinnati, OH, USA) was used at a dilution of 1:250 in Ventana antibody diluent with casein (760-219, Roche Diagnostics) and incubated at 37 °C for 1 h. Signals were developed with chromogen Discovery yellow HRP (760-250, Roche Diagnostics).

Digitalized whole slide images (WSI) of the IHC were generated using a multispectral PhenoImager HT (AKOYA Biosciences, Marlborough, MA, USA). IHC-stained slides were scanned at 200× total magnification in a bright field. Images were generated by the software and stored in qptiff format. Quantification of the IHC signals was achieved by using the HALO image analysis software v4 (Indica labs, Albuquerque, NM, USA). Each image was annotated by a board-certified pathologist (ML) to define the regions of interest. The total examined pulmonary parenchyma with associated airways in all the sections on the entire selected slide were included in the region of interest and non-tissue areas or non-pulmonary tissues were excluded to ensure the calculation of the percentage of the areas with IHC signals reflect the total examined pulmonary parenchyma. Pulmonary carbon pigments and non-specific signals of cartilage, and luminal or acellular debris were excluded from the region of interest manually. Indica Labs-Area Quantification v2.4.9 algorithm provided in the HALO was applied to define the areas of positive IHC signals. The same quantifying algorithm was applied to all four subjects to yield the quantification results.

## 3. Results

### 3.1. Morbidity and Mortality

All four animals survived to the end of the study (5 dpi) and were humanely euthanized. None of the four animals showed any observable signs of illness for the duration of the study, which is consistent with previous reports of SARS-CoV-2 disease in the AGM [8,9,16].

### 3.2. Body Weights

**Figure 1:**
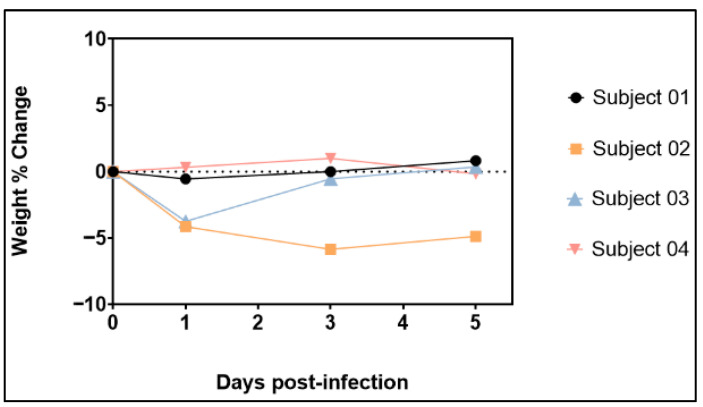
Percent Body Weight Change in Animals (two females, Subject 01 and 02, and two males, Subject 03 and 04) Exposed to SARS-CoV-2 Omicron XBB.1.5 variant.

Body weights were collected at 0, 1, 3 and 5 dpi. The percentage change in body weight was determined using 0 dpi as the baseline. Body weight remained relatively stable in the majority of animals, with only one (Subject 02, female) experiencing a weight loss of more than five percent (5.9%) at 3 dpi (Figure 1) and not returning to baseline levels by the end of the study. At 1 dpi, Subjects 02 and 03 experienced a slight weight loss of 4.1% and 3.7%, respectively. These early changes in body weight are considered inconsequential and are likely due to the animals limiting food intake following sedation for viral exposure. There was no association between changes in body weight and sex.

### 3.3. Viral Burden

Viral burden in tissues, serum, and BALF was determined using Vero E6 cells and a crystal violet plaque assay. In tissues, infectious virus was detected in a single lung tissue sample for one animal (Subject 02, female), with a titer of 1 160 PFU/gram (Table S1). The detection of infectious virus corresponds to the decrease in body weight seen for this animal. Infectious virus was only detected in one BALF sample of a single subject (Subject 04, male) on 5 dpi, with a titer of 220 PFU/mL (Table S2). Consistent with previous reports, infectious virus could not be detected in any of the serum samples tested [9,16].

**Figure 2:**
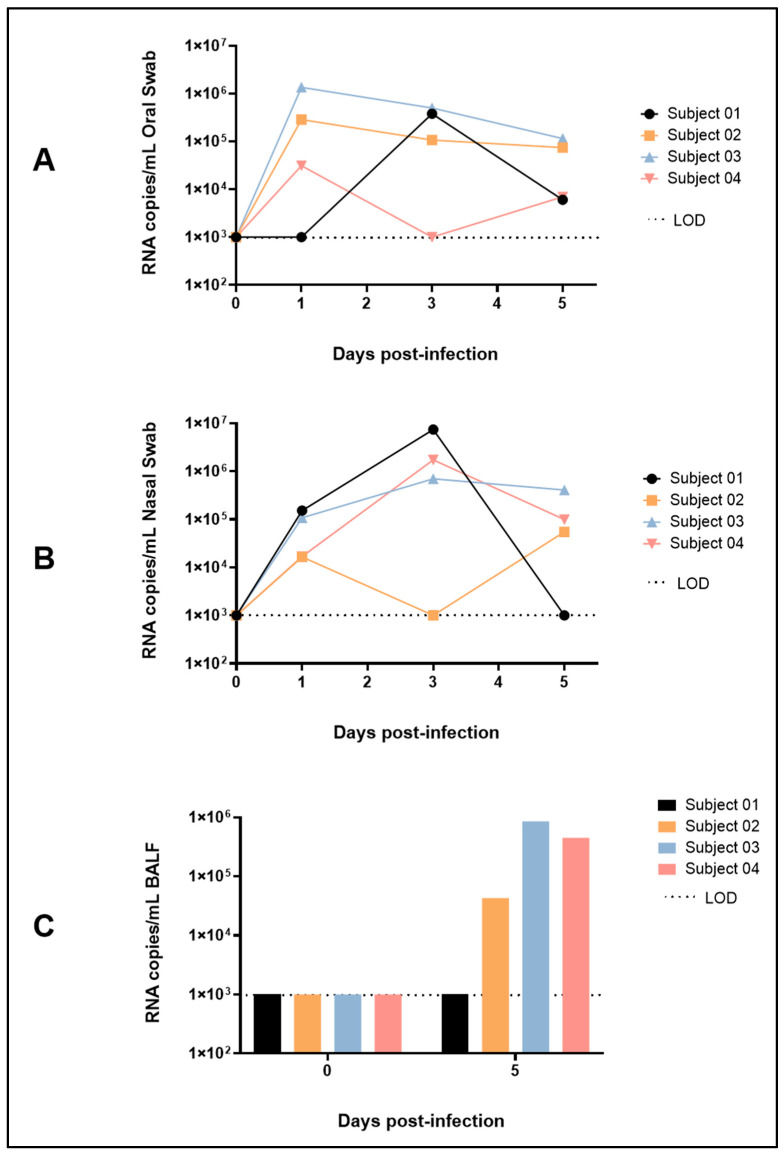
Quantitative Reverse Transcription Polymerase Chain Reaction Results in (**A**) Oral Swabs, (**B**) Nasal Swabs and (**C**) Bronchoalveolar Lavage Fluid for Animals Exposed to SARS-CoV-2 Omicron XBB.1.5 variant. LOD = limit of detection.

A sub-genomic RT-qPCR assay targeting the E gene of SARS-CoV-2 was used to detect the presence of viral replicative RNA intermediates in tissues, serum, swabs and BALF. No SARS-CoV-2 RNA was detected by RT-qPCR in the serum of any of the animals for the duration of the study. Figure 2 shows the RT-qPCR results for oral swabs, nasal swabs and BALF. Viral RNA levels peaked in oral swabs for three of the four animals at 1 dpi, reaching concentrations of up to 1.35 × 10^6^ RNA copies/mL (Figure 2A). In nasal swabs, viral RNA levels peaked at 3 dpi for three of the four animals, reaching concentrations up to 7.38 × 10^6^ RNA copies/mL (Figure 2B). All but one animal had detectable viral RNA in BALF samples taken 5 dpi, with concentrations reaching up to 8.55 × 10^5^ RNA copies/mL (Figure 2C).

Tissue RT-qPCR data are shown in Table 1. Subject 01 and 02 (both females), each had a single tissue test positive for viral RNA, while viral RNA detection was more dispersed in the two males. All animals except Subject 01 had detectable viral RNA in the nasal turbinates at the end of the study (5 dpi).

### 3.4. Body Temperature

**Figure 3:**
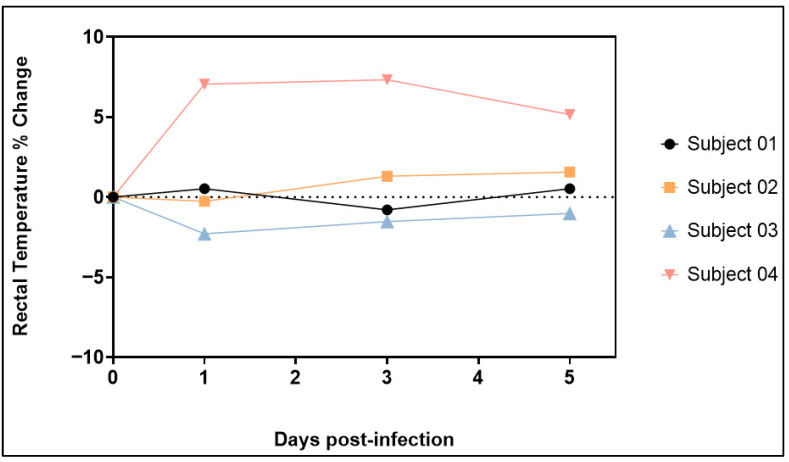
Percent Temperature Change in Animals Exposed to SARS-CoV-2 XBB.1.5.

Rectal temperatures were collected at 0, 1, 3 and 5 dpi. The percentage change in body temperature was determined using 0 dpi as the baseline. Body temperatures remained relatively stable for the duration of the study in the majority of animals. One animal (Subject 04, male) experienced an increase in body temperature of up to 7.34% between 1 and 5 dpi (Figure 3), with temperatures ranging from 36.8 °C to 39.5 °C, indicating fever.

### 3.5. Hematology

**Figure 4:**
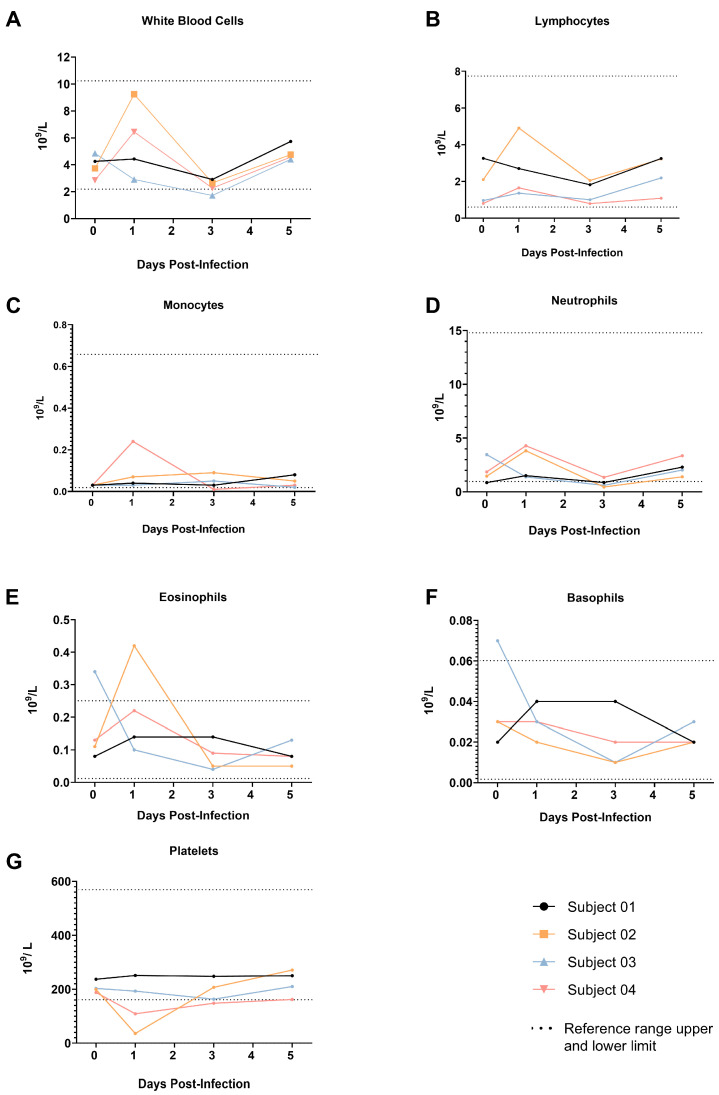
Change in Hematology Parameters of Animals Exposed to SARS-CoV-2 XBB.1.5. (**A**) White blood cells, (**B**) Lymphocytes, (**C**) Monocytes, (**D**) Neutrophils, (**E**) Eosinophils, (**F**) Basophils, (**G**) Platelets.

Hematological analyses were performed using the Abaxis VetScan HM5 (Zoetis, Parsippany, NJ, USA) analyzer. The majority of parameters were within or only slightly outside of the published normal reference range [17]. A mild elevation of absolute numbers of eosinophils at 1 dpi in Subject 02 (Figure 4) was noted. Platelets were mildly reduced in two animals (Subjects 02 and 04) at 1 dpi, indicating mild thrombocytopenia similar to the mild thrombocytopenia reported in a minor subset of humans infected with SARS-CoV-2 that are mostly non-ICU cases [18]. No noteworthy changes were noted in any of the other parameters tested.

### 3.6. Clinical Chemistry

**Figure 5:**
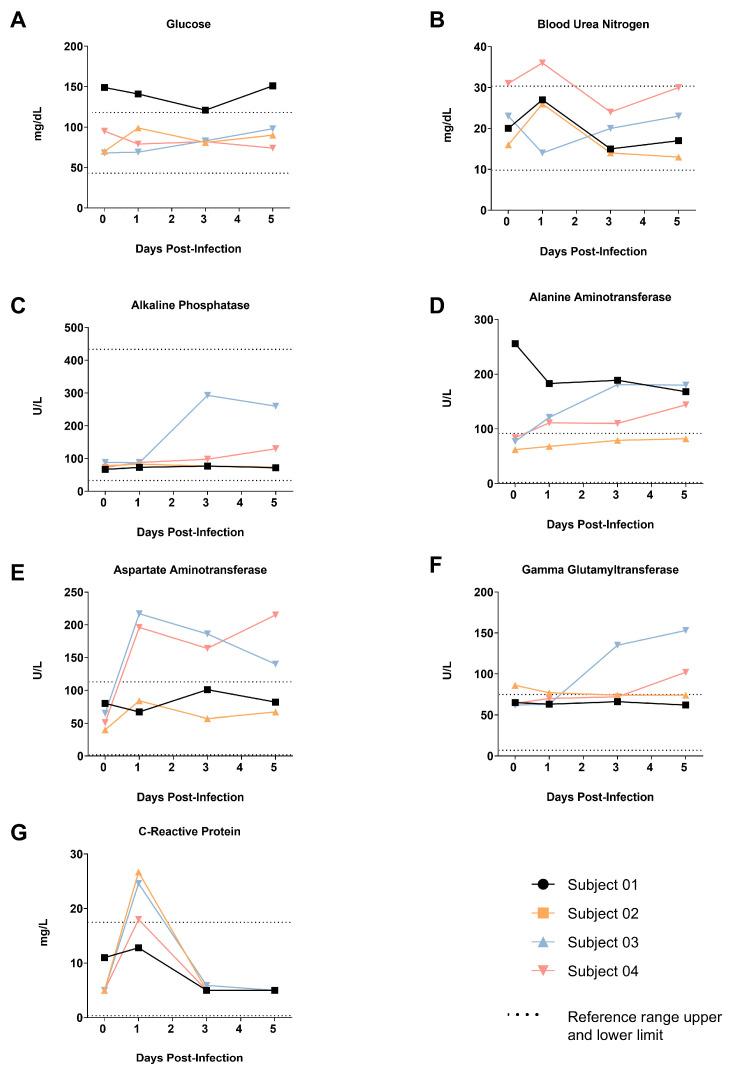
Change in Clinical Chemistry Parameters of Animals Exposed to SARS-CoV-2 Omicron XBB.1.5. (**A**) Glucose, (**B**) Blood Urea Nitrogen, (**C**) Alkaline Phosphatase, (**D**) Alanine Aminotransferase, (**E**) Aspartate Aminotransferase, (**F**) Gamma Glutamyltransferase, (**G**) C-Reactive Protein.

Clinical chemistry testing was performed using the Abaxis Biochemistry Panel Plus rotor (Zoetis, Parsippany, NJ, USA). Results are shown in Figure 5. In all animals except Subject 01, C-reactive protein was elevated by 1 dpi, consistent with acute systemic inflammation brought on by infection with SARS-CoV-2. Markers for hepatic function increased to beyond the published normal reference range [17] for both males (Subjects 03 and 04) from 1 dpi (alanine aminotransferase, aspartate aminotransferase) or 3 dpi (gamma glutamyltransferase) to study termination, consistent with more severe disease in the males compared to the females. Changes in other clinical chemistry parameters tested were clinically unremarkable.

### 3.7. Cytokine and Chemokine Expression

**Figure 6:**
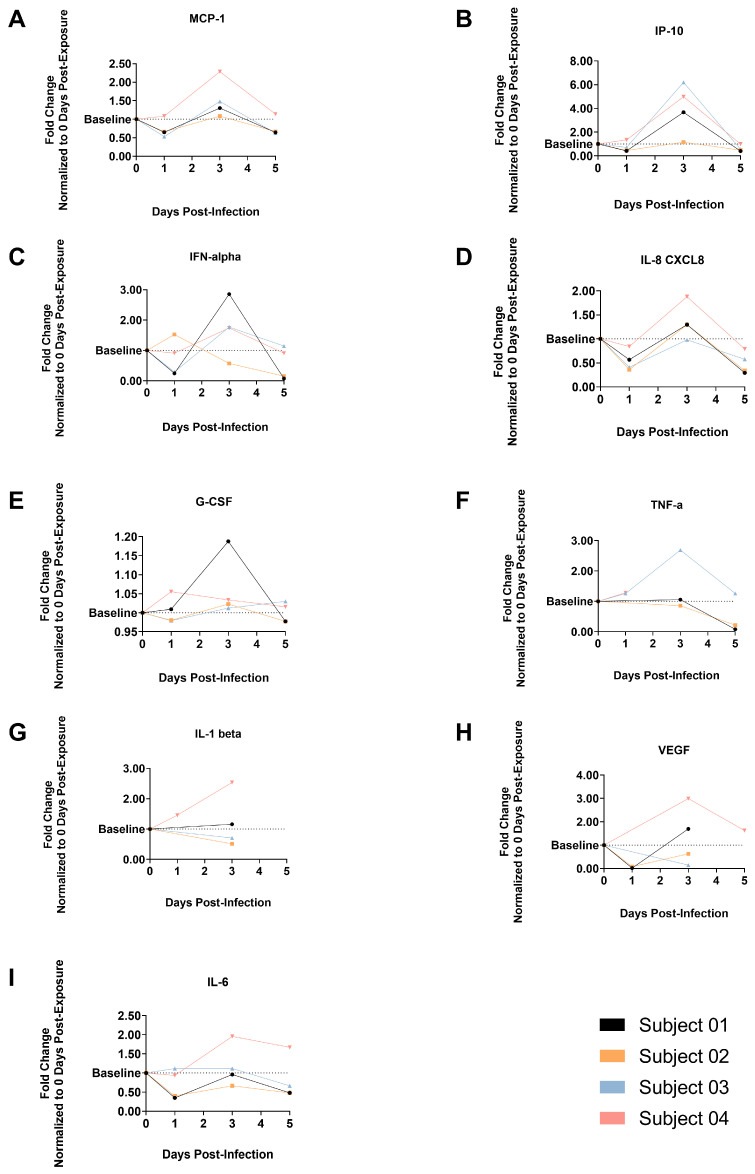
Fold Change from Baseline in Cytokines and Chemokines in Plasma for Animals Exposed to SARS-CoV-2 XBB.1.5. (**A**) Monocyte Chemoattractant Protein-1, (**B**) C-X-C motif chemokine 10 (IP10), (**C**) Interferon Alpha, (**D**) Interleukin 8, (**E**) Granulocyte Colony-Stimulating Factor, (**F**) Tumor Necrosis Factor Alpha, (**G**) Interleukin 1 Beta, (**H**) Vascular Endothelial Growth Factor, (**I**) Interleukin 6.

The cytokine and chemokine levels in plasma and BALF were determined using Luminex xMAP technology (Bio-Techne, Corporation, Minneapolis, MN, USA). The changes in protein levels for MCP-1, IP-10, G-CSF, IFN-a, IFN-b, IFN-g, IL-1b, IL-12, IL-8, TNF-a, VEGF and IL-6 were analyzed using 0 dpi as the baseline. Figure 6 shows the protein expression changes normalized against the baseline of the indicated analytes in plasma and BALF. In plasma, three of the four animals (Subjects 01, 03 and 04) showed an increase in MCP-1, IP-10 and IFN-a 3 dpi, indicating an efficient immune response to active SARS-CoV-2 replication [19] and subsequent clearance of infectious virus from the blood by 5 dpi. Plasma samples from Subject 01 also showed an increase in G-CSF expression, Subject 03 showed an increase in TNF-a and Subject 04 showed an increase in TNF-a, IL-1b, VEGF and IL-16 (Figure 6). There were no notable changes in the IFN-b and IL-12 concentration in the plasma for any of the animals for the duration of the study. Of interest, infectious virus was only detected in lung samples from Subject 02, which is indicative of the poor immune response generated in the early phases of infection that subsequently allowed the virus to disseminate to the lower respiratory tract.

**Figure 7:**
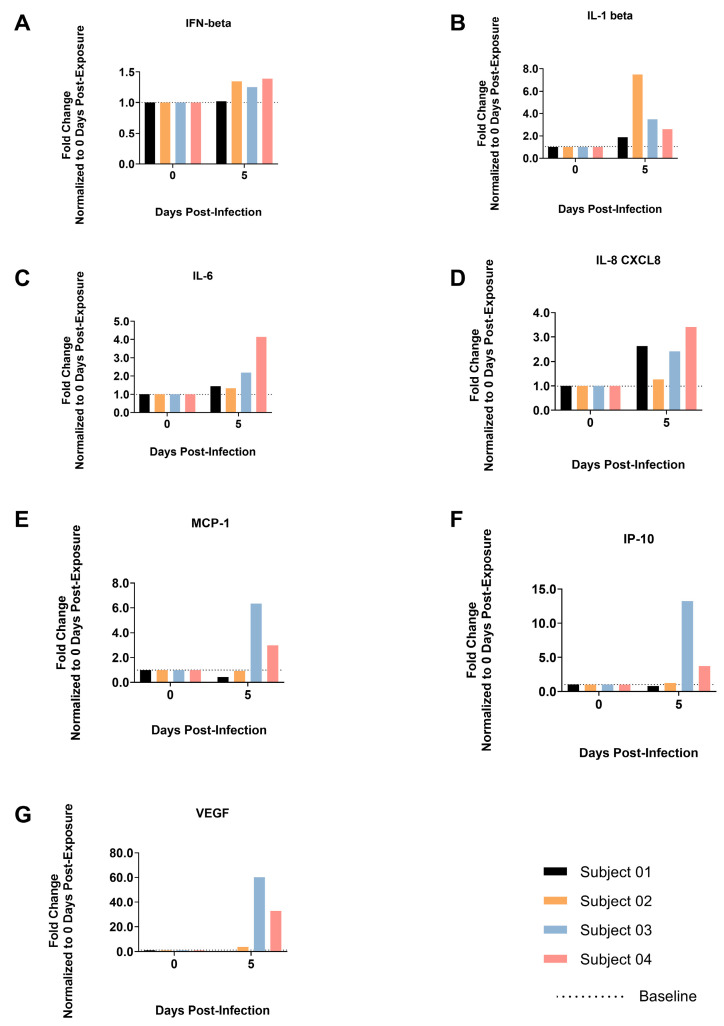
Fold Change from Baseline in Cytokines and Chemokines in Bronchoalveolar Lavage Fluid for Animals Exposed to SARS-CoV-2 XBB.1.5. (**A**) Interferon Beta, (**B**) Interleukin 1 Beta, (**C**) Interleukin 6, (**D**) Interleukin 8, (**E**) Monocyte Chemoattractant Protein-1, (**F**) C-X-C motif chemokine 10 (IP10), (**G**) Vascular Endothelial Growth Factor.

IFN-b, IL-1b, IL-6 and IL-8 expression in BALF samples 5 dpi showed a noteworthy increase in expression in all animals but Subject 01, which showed no or only a limited increase in these analytes with the exception of IL-8. MCP-1, IP-10 and VEGF expressions were also increased in Subjects 03 and 04 (Figure 7), with IP-10 being the most consistently elevated marker in all animals. There were no notable changes in IFN-a, IFN-g, TNF-a, G-CSF and IL-12 levels in BALF samples. These data are consistent with an acute phase immune response in the lower respiratory tract of these animals, correlating with the presence of viral RNA and infectious virus detected in the lung and/or BALF samples of Subjects 03 and 04.

### 3.8. Gross Pathology and Histopathology Findings

**Figure 8:**
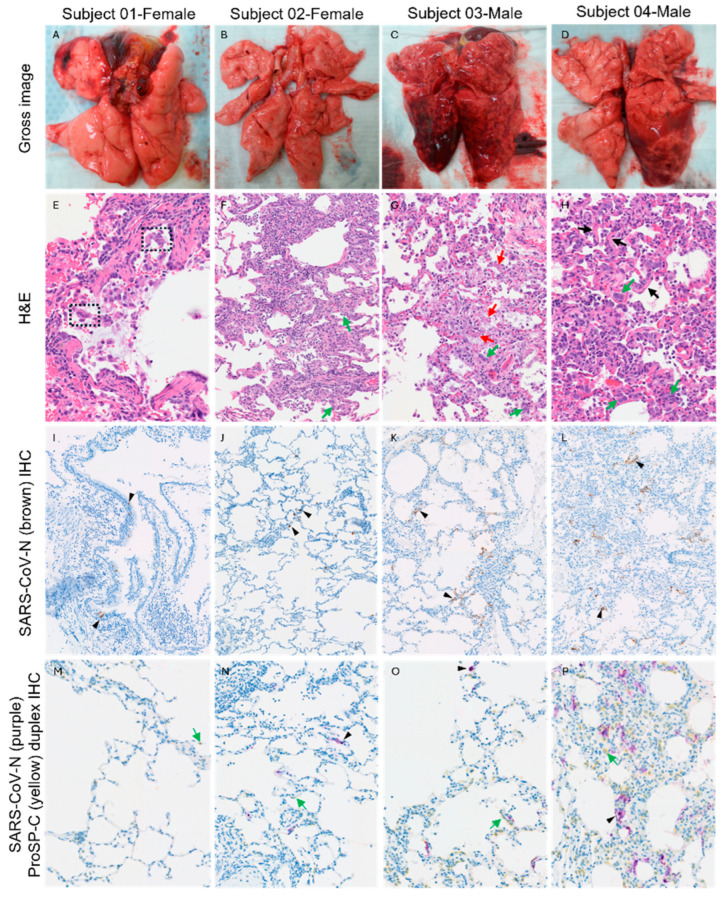
Gross and Histopathologic Findings in Animals Exposed to SARS-CoV-2 XBB.1.5. (**A**–**D**): Gross pathology. (**E**–**H**): Histopathologic findings including syncytial cells (**E**-black hashed boxes), mononuclear and neutrophilic alveolar interstitial infiltrates (**F**–**H**), alveolar fibrin deposits (**G**-red arrows), hyaline membranes (**H**-black arrows), and AT2 pneumocyte hyperplasia (**F**–**H**-green arrows). (**I**–**L**): Positive IHC signals of SARS-CoV N protein (arrowhead) in rare bronchial epithelial cells (**I**), distal lung and alveoli (**J**–**L**). (**M**): A representative Type 2 pneumocyte that is within normal limits expressing positive (yellow) IHC signals of ProSP-C (green arrow). (**N**–**P**): Minimal (**N**,**O**) to moderate numbers (**P**) of hyperplastic AT2 pneumocytes identified by their cuboidal shape and yellow ProSP-C IHC signals (green arrows) in areas of pneumonia with SARS-CoV-2 N protein signals (purple, arrowhead). (**E**,**I**,**M**): Right caudal lung lobe, Subject 01. (**F**,**J**,**N**): Left cranial lung lobe, Subject 02. (**G**,**K**,**O**): Left cranial lung lobe, Subject 03. (**H**,**L**,**P**): Left cranial lung lobe, Subject 04.

**Figure 9:**
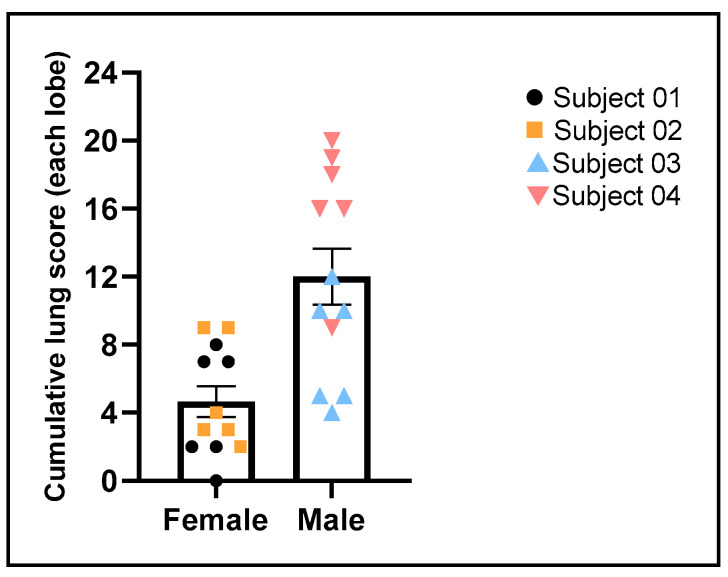
Cumulative lung ordinal scores of individual lung lobe from each animal.

The most significant pathological findings observed in this study were in the lungs. Gross lesions characterized by failure to collapse and parenchymal reddening of all lung lobes were observed in Subjects 03 and 04, while the lungs in Subjects 01 and 02 were within normal limits, with minimal parenchymal reddening attributable to euthanasia-associated perimortem pulmonary hemorrhage observed in Subject 01 (Figure 8A,D). Microscopically, all animals showed minimal to marked mononuclear and neutrophilic bronchiolitis, attributable to SARS-CoV-2 infection. Histopathological hallmarks of more advanced pulmonary lesions involving distal lungs and alveoli consistent with SARS-CoV-2 infection included mild to marked lymphohistiocytic and neutrophilic bronchointerstitial pneumonia that centered on terminal bronchioles, with evidence of acute lung injury and repair, such as alveolar septal necrosis or hyaline membranes, alveolar fibrin deposits, and AT2 pneumocyte hyperplasia were detected in Subjects 02–04 (Figure 8E–H,N–P; Table S4), but not in Subject 01 (Figure 8M). Among these three animals, Subject 04 displayed the most severe pneumonia with a total lung ordinal score of 98/144 (cumulative lung ordinal score 9–20/24 of each lung lobe, Table S4, Figure 9). In all lung lobes of Subject 04, there was a median of moderate to marked bronchiole-centric bronchointerstitial pneumonia, neutrophilic and lymphohistiocytic, multifocal to coalescing, with moderate AT2 hyperplasia, occasional septal necrosis, and hyaline membrane formation, rare intra-alveolar fibrin aggregates, and mild mononuclear perivascular infiltrates (Figure 8H,P, Table S4). Subject 03 showed similar but less severe bronchiole-centric bronchointerstitial pneumonia compared to Subject 04, with a total lung ordinal score of 46/144 (cumulative lung ordinal score 4–12/24 of each lung lobe, Table S4, Figure 9). In all lung lobes of Subject 03, there was a median of mild to moderate lymphohistiocytic and neutrophilic bronchiole-centric bronchointerstitial pneumonia, multifocal to coalescing, with minimal AT2 hyperplasia, rare septal necrosis, rare intra-alveolar fibrin aggregates, and mild mononuclear perivascular infiltrates (Figure 8G,O, Table S4). Compared to the males, the only female among these three animals (Subject 02) had the mildest bronchointerstitial pneumonia that restricted to only left lobes, with a total lung ordinal score of 30/144 (cumulative lung ordinal score 2–9/24 of each lung lobe, Table S4, Figure 9). In the left cranial and left caudal lung lobes of Subject 02, there was minimal to mild, multifocal lymphohistiocytic bronchiole-centric bronchointerstitial pneumonia associated with minimal AT2 hyperplasia, minimal to mild perivascular cuffing, and rare septal necrosis (Figure 8F,N, Table S4). Conversely, Subject 01 presented predominantly with bronchiolitis without significant alveolar damage and yielded the lowest total lung ordinal score of 26/144 (cumulative lung ordinal score 0–7/24 of each lung lobes, Table S4, Figure 9). However, rare syncytial cells were detected in affected bronchioles in Subject 01 (Figure 8E). In summary, while the pulmonary lesions were variable in severity, all infected animals had histopathological findings attributable to SARS-CoV-2 infection; and males tend to develop more advanced lymphohistiocytic and neutrophilic, bronchiole-centric bronchointerstitial pneumonia with evidence of acute lung injury and repair with higher average cumulative lung ordinal scores compared to females (Figure 9). While bronchoalveolar lavage can induce transient alveolar neutrophilia, this effect is typically short-lived and was minimized in our study by limited BAL procedures spaced widely apart. Furthermore, the key pathological features observed—such as delayed AT2 hyperplasia and lymphocytic infiltrates—are unlikely to result from BAL and instead reflect genuine SARS-CoV-2–induced lung injury. Therefore, we do not consider BAL-related artifacts to be a significant confounding factor in our histopathologic analysis.

**Figure 10:**
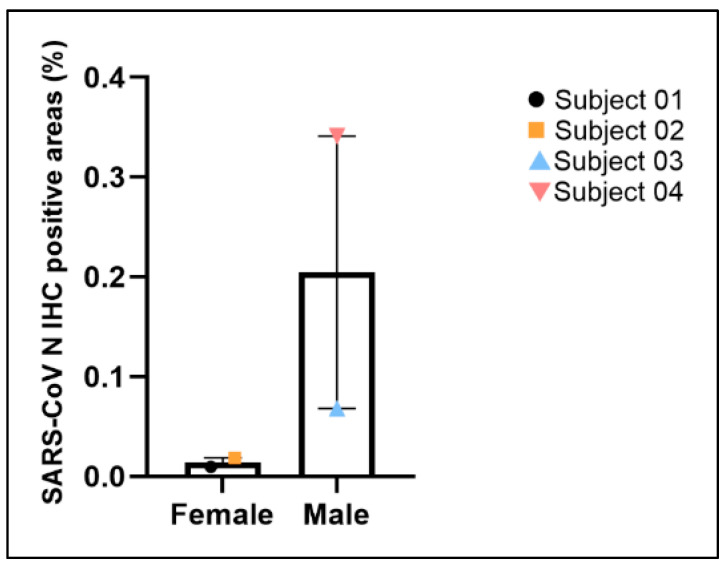
Quantification of the percentage of SARS-CoV-N IHC positive areas in the whole slide of selected lung lobe from each animal.

IHC staining of SARS-CoV N protein identified positive signals in the representative lung sections of all four animals, confirming SARS-CoV-2 infection of all subjects (Figure 8I–L). In Subject 01, SARS-CoV N-positive signals were detected in rare bronchial epithelial cells (Figure 8I) but were absent in distal lung and alveolar septa, correlating to the histopathological findings of bronchiolitis without alveolar damage. In Subject 02, SARS-CoV-N-positive signals were detected in rare bronchial epithelial cells and occasional multifocal alveolar septa (Figure 8J). In Subjects 03 and 04, SARS-CoV-2-N-positive signals were predominantly observed in distal lung and alveolar septa in multifocal to coalescing areas (Figure 8K,L), in addition to in rare bronchial epithelial cells. Digital quantification of the IHC positive areas in the whole slide of the representative lung lobe in each subject revealed the same trend that precisely correlated with the cumulative lung ordinal score where males displayed more SARS-CoV N positivity in tissue than females (Figure 9 and Figure 10). Subject 04 displayed the most SARS-CoV N-positive areas followed by Subject 03. Subject 01 had the least SARS-CoV N-positive areas. Overall, the percentages of SARS-CoV N-positive areas of the entire regions of interest (entire examined pulmonary parenchyma of the whole slide of the selected lung lobe) were less than 0.4%, with 0.0095%, 0.0185%, 0.068%, and 0.34% for Subject 01, 02, 03 and 04, respectively. The amounts of SARS-CoV N IHC immunopositivity corresponded to the severity of lung lesions (Figure 9 and Figure 10). Specifically, there were more SARS-CoV N IHC immunoreactivities in animals with more severe lung lesions. Duplex IHC for SARS-CoV N and ProSP-C confirmed the histological interpretation of minimal (Subjects 02 & 03) to moderate (Subject 04) AT2 pneumocyte hyperplasia in SARS-CoV N-associated pneumonia (Figure 8N,P). Rare, individual cells (possibly dendritic cells or macrophages based on the cell shape) expressed mild SARS-CoV N signals in the trabecular or medullary sinuses of lymph nodes of all subjects.

## 4. Discussion

Four AGMs were exposed IN to the SARS-CoV-2 Omicron XBB.1.5 subvariant. All animals survived to the end of the study, with no observable signs of illness and limited weight loss consistent with the mild-to-moderate disease phenotype observed in humans infected with this subvariant [20]. Fever was only observed in a single animal, which corresponded with the most advanced bronchiole-centric, bronchointerstitial pneumonia in this animal among this cohort. Viremia was absent in this study, and detection of infectious virus by plaque assay in BALF and tissues was limited, suggesting a robust antiviral response and rapid viral clearance following initial infection [21]. However, the presence of subgenomic SARS-CoV-2 RNA in swabs, BALF and certain tissues, most notably in the two males in the study, may suggest continued viral replication or persistence [22]. Changes in hematological and clinical chemistry markers were unremarkable and primarily associated with acute systemic inflammation shortly following exposure to SARS-CoV-2, such as C-reactive protein. These early transient inflammatory responses are typical of viral infections but did not result in prolonged clinical manifestations in this model [23]. At 1 dpi, the mild eosinophilia in Subject 02; and the minimally increased absolute numbers of eosinophils compared to their own baseline in Subject 01 and 04 may or may not be associated with the moderate hypereosinophilic bronchitis interpreted as hypersensitivity of unknown origin at the termination of study (5 dpi). The exclusion of large conducting airways in the lung ordinal scoring criteria ensures avoiding potential confounding factors from the concurrent eosinophilic bronchitis. Histologically, all animals developed bronchiolitis with variable numbers of syncytial cells attributable to SARS-CoV-2 infection, which is a hallmark of the virus’s ability to disrupt the respiratory epithelium [24]. Positive SARS-CoV N IHC signals were detected the lung and tracheobronchial lymph nodes in all animals, confirming infection of all subjects. In Subject 01, the pulmonary lesions attributable to viral infection were limited to mild bronchiolitis without significant alveolar involvement, supported by limited SARS-CoV-N IHC signals, viral detection and cytokine/chemokine responses in this animal. In contrast, more advanced pulmonary lesions that extended to the distal lung, as characterized by terminal bronchiole-centric bronchointerstitial pneumonia with evidence of alveolar injury and type 2 pneumocyte hyperplasia, were detected in the two male and one of the female animals. The males displayed more severe gross and histopathological lesions and more SARS-CoV N IHC immunoreactivities compared to the females in this small cohort. The more severe pneumonia in the males than the female correlated with the higher viral RNA burden in the BALF collected on the day of necropsy in the males (Subjects 03 and 04) followed by Subject 02. Infectious virus was detected in the BALF of Subject 04, in which the animal had the most severe pneumonia and the most abundant SARS-CoV N IHC immunoreactivities in the lung. The most significant elevation of plasma proinflammatory cytokines (IL-6, IL-1β, MCP-1, IP-10 and TNF-α) at 5 dpi were also detected in Subjects 03 and/or 04, suggesting more severe pneumonia-induced systemic inflammation [25,26]. Furthermore, hepatic clinical chemistry markers including alanine aminotransferase, aspartate aminotransferase and gamma glutamyltransferase were more pronouncedly elevated in the males. These findings suggest a possible sex-based difference in disease severity caused by SARS-CoV-2 XBB.1.5, and aligns with previous reports in animal models and human cohorts which indicated that the male sex may be associated with more severe COVID-19 outcomes, possibly due to differences in immune responses [27,28,29].

This study was not without limitations. Firstly, the number of animals used in this study was constrained by ethical and logistical considerations consistent with exploratory studies in nonhuman primates; therefore, statistical analysis of the results was not supported, and the findings should be interpreted with caution. The sex and disease correlation especially warrants further investigation. Second, the absence of control groups, including uninfected controls or animals infected with other variants of SARS-CoV-2, precluded direct comparative analysis and limited the ability to contextualize the observed disease manifestations. Thirdly, while intranasal instillation ensures reproducible dosing and comparability with prior coronavirus studies, it does not fully recapitulate natural infection. Future studies will employ inhalational exposure with aerosolized virus to better mimic the natural route of transmission. Finally, although beyond the scope of this study, the limited duration of the study prohibited assessment of convalescence or long-term outcomes. Future studies will address these limitations by including comparative cohorts infected with other SARS-CoV-2 variants, including Alpha, to enable direct comparisons of disease severity and immune responses. Furthermore, the observation period will be extended beyond 5 days post-infection to assess recovery trajectories, viral persistence, and long-term outcomes.

## 5. Conclusions

Our results indicate that AGMs exposed to SARS-CoV-2 XBB.1.5 become infected and exhibit a mild to moderate, self-limiting disease phenotype without clinically significant symptoms, mirroring observations in humans infected with this variant. Acutely infected AGMs display histological evidence of bronchiolitis to bronchointerstitial pneumonia of variable severity. Whilst our study was limited in scope and preliminary in nature, our findings broaden the understanding of SARS-CoV-2 pathogenesis in the AGM IN model of disease. With further study, this model could hold promise for studying viral pathogenesis, host immune responses, and evaluating the efficacy of potential medical interventions against newer variants of SARS-CoV-2 and could be a valuable addition to existing models focused on earlier variants.

## Figures and Tables

**Table 1:** Quantitative Reverse Transcription Polymerase Chain Reaction Results in Tissues Collected Five Days Post-Infection from Animals Exposed to SARS-CoV-2 XBB.1.5.

	RNA Copies Per Gram Tissue ^1^			
Tissue	Subject 01	Subject 02	Subject 03	Subject 04
Right cranial lung	BLD	BLD	BLD	BLD
Right middle/caudal lung	BLD	BLD	BLD	BLD
Accessory lung	BLD	BLD	1.33 × 10^6^	BLD
Left cranial lung	BLD	BLD	3.88 × 10^7^	BLD
Left caudal lung	BLD	BLD	BLD	BLD
Tracheobronchial lymph node	BLD	BLD	BLD	3.38 × 10^4^
Brain frontal lobe	BLD	BLD	BLD	BLD
Cerebellum	BLD	BLD	BLD	BLD
Brainstem	BLD	BLD	BLD	BLD
Olfactory bulb	BLD	BLD	BLD	BLD
Nasal turbinate	BLD	1.90 × 10^5^	1.85 × 10^6^	1.85 5
Tonsil	BLD	BLD	BLD	6.35 × 10^4^
Spleen	BLD	BLD	BLD	BLD
Liver	BLD	BLD	BLD	BLD
Colon	1.93 × 10^7^	BLD	BLD	BLD
Submandibular lymph node	BLD	BLD	BLD	BLD

^1^ BLD—below limit of detection.

## Data Availability

The original contributions presented in this study are included in the article/Supplementary Material. Further inquiries can be directed to the corresponding author.

## Supplementary Materials

The following supporting information can be downloaded at: https://www.mdpi.com/article/10.3390/v17101373/s1, Table S1: Infectious Virus as Measured by Plaque Assay in Tissues Collected from Animals Exposed to SARS-CoV-2 XBB.1.5; Table S2: Infectious Virus as Measured by Plaque Assay in Serum and Bronchoalveolar Lavage Fluid Collected from Animals Exposed to SARS-CoV-2 XBB.1.5; Table S3: Lung Ordinal Scoring Criteria; Table S4: Individual Animal Lung Ordinal Scores.

## Abbreviations

The following abbreviations are used in this manuscript:

AAALAC	Association for Assessment and Accreditation of Laboratory Animal Care
ABLS-4	Animal biosafety level 4
ACURO	Animal Care and Use Review Office
AGM	African green monkey
ARDS	Acute respiratory distress syndrome
AT2	Alveolar type 2
BALF	Bronchioalveolar lavage fluid
COVID-19	Coronavirus disease 2019
CXCL10	C-X-C motif chemokine 10
DAB	3,3′-diaminobenzidine
dpi	Days post-infection
DMEM	Dulbecco’s Modified Eagle Medium
EDTA	Ethylenediaminetetraacetic acid
FFPE	Formalin fixed paraffin embedded
G-CSF	Granulocyte colony-stimulating factor
IACUC	Institutional Animal Care and Use Committee
IFN	Interferon
IHC	Immunohistochemistry
IL	Interleukin
IN	Intranasal
MCP-1	Monocyte chemoattractant protein-1
NHP	Nonhuman primate
PBS	Phosphate-buffered saline
PFU	Plaque forming units
RT-qPCR	Quantitative reverse transcription polymerase chain reaction
SARS-CoV-2	Severe acute respiratory syndrome coronavirus 2
SIV	Simian immunodeficiency virus
SRV	Simian retrovirus
STLV-1	Simian T-lymphotrophic virus-1
TNF	Tumor necrosis factor
USAMRAA	U.S. Army Medical Research Acquisition Activity
VEGF	Vascular endothelial growth factor
