# Supplementary material for: An Intranasal Challenge Model in African Green Monkeys (Chlorocebus aethiops) for Mild-to-Moderate COVID-19 Disease Caused by Subvariant XBB.1.5
Source: Viruses. 2025 Oct 14;17(10):1373. doi: 10.3390/v17101373 (PMC12568310; doi:10.3390/v17101373)
Supplement: Supplementary file 1 [file viruses-17-01373-s001.zip › Table S1 - Infectious Virus as Measured by Plaque Assay in Tissues.pdf]

Table S1. Infectious Virus as Measured by Plaque Assay in Tissues Collected from Animals Exposed to SARS-CoV-2 XBB.1.5

	**Plaque forming units per gram tissue**			
**Tissue**	**Subject 01**	**Subject 02**	**Subject 03**	**Subject 04**
**Right cranial lung**	ND	ND	ND	ND
**Right middle/caudal lung**	ND	1 160	ND	ND
**Accessory lung**	ND	ND	ND	ND
**Left cranial lung**	ND	ND	ND	ND
**Left caudal lung**	ND	ND	ND	ND
**Tracheobronchial lymph node**	ND	ND	ND	ND
**Brain frontal lobe**	ND	ND	ND	ND
**Cerebellum**	ND	ND	ND	ND
**Brainstem**	ND	ND	ND	ND
**Olfactory bulb**	ND	ND	ND	ND
**Nasal turbinate**	ND	ND	ND	ND
**Tonsil**	ND	ND	ND	ND
**Spleen**	ND	ND	ND	ND
**Liver**	ND	ND	ND	ND
**Colon**	ND	ND	ND	ND
**Submandibular lymph node**	ND	ND	ND	ND

Abbreviations: ND – not detected
