# Supplementary material for: An Intranasal Challenge Model in African Green Monkeys (Chlorocebus aethiops) for Mild-to-Moderate COVID-19 Disease Caused by Subvariant XBB.1.5
Source: Viruses. 2025 Oct 14;17(10):1373. doi: 10.3390/v17101373 (PMC12568310; doi:10.3390/v17101373)
Supplement: Supplementary file 1 [file viruses-17-01373-s001.zip › Table S2 - Infectious Virus as Measured by Plaque Assay in Serum and BALF.pdf]

Table S2. Infectious Virus as Measured by Plaque Assay in Serum and Bronchoalveolar Lavage Fluid Collected from Animals Exposed to SARS-CoV-2 XBB.1.5

	Bronchoalveolar lavage fluid (PFU/mL)		Serum (PFU/mL)			
**Animal ID**	**Study Day 1**	**Study Day 6**	**Study Day 1**	**Study Day 2**	**Study Day 4**	**Study Day 6**
01	ND	ND	ND	ND	ND	ND
02	ND	ND	N/A	ND	ND	ND
03	ND	ND	ND	ND	ND	ND
04	ND	220	ND	ND	ND	ND

Abbreviations: N/A – not applicable as insufficient sample was collected; ND – not detected.
