# Supplementary material for: An Intranasal Challenge Model in African Green Monkeys (Chlorocebus aethiops) for Mild-to-Moderate COVID-19 Disease Caused by Subvariant XBB.1.5
Source: Viruses. 2025 Oct 14;17(10):1373. doi: 10.3390/v17101373 (PMC12568310; doi:10.3390/v17101373)
Supplement: Supplementary file 1 [file viruses-17-01373-s001.zip › Table S3 - Lung Ordinal Scoring Criteria.pdf]

Table S3. Lung Ordinal Scoring Criteria	
**% of lung area affected**	
0	No significant findings.
1	<10%
2	11-30 %
3	31-50 %
4	>50%
	**Neutrophilic and lymphohistiocytic inflammation in terminal bronchioles**
0	No significant findings.
1-minimal	Presence of luminal exudate and/or inflammatory cell infiltration +/- multinucleated giant cells and syncytial cells; rarely observed.
2-mild	Presence of luminal exudate and/or inflammatory cell infiltration +/- multinucleated giant cells and syncytial cells; up to 30% of bronchioles involved.
3moderate	Presence of luminal exudate and/or inflammatory cell infiltration +/- multinucleated giant cells and syncytial cells; 31-50% of bronchioles involved.
4- marked to severe	Presence of luminal exudate and/or inflammatory cell infiltration +/- multinucleated giant cells and syncytial cells; >50% of bronchioles involved.
	**Inflammation in the alveolar spaces**
1-minimal	Presence of inflammatory cell infiltration; up to 10% observed.
2-mild	Presence of inflammatory cell infiltration; 11-30 % of alveolar spaces occupied
3moderate	Presence of inflammatory cell infiltration; 31-50% of alveolar spaces occupied.
4-marked to severe	Presence of luminal exudate and/or inflammatory cell infiltration; over 50% alveolar spaces occupied.
**Alveolar septal pneumocyte necrosis and repair, +/- hyaline membranes and syncytial cells**	
0-normal	No findings.
1-minimal	Alveolar type II (AT2) pneumocyte hyperplasia, +/- septal necrosis or hyaline membrane and/or syncytial cells; up to 10% of the slide affected.
2-mild	Alveolar type II (AT2) pneumocyte hyperplasia, +/- septal necrosis or hyaline membrane and/or syncytial cells; 11-30% of the slide affected.
3- moderate	Alveolar type II (AT2) pneumocyte hyperplasia, +/- septal necrosis or hyaline membrane and/or syncytial cells; 31-50% of the slide affected.
4- marked to severe	Alveolar type II (AT2) pneumocyte hyperplasia, +/- hyaline membrane and/or syncytial cells; >50% of the slide affected.
**Alveolar interstitial inflammation**	
0-normal	No findings.
1-minimal	Thickening of alveolar walls by mixed inflammatory cells; up to 10% of the slide affected.
2-mild	Thickening of alveolar walls by mixed inflammatory cells; 11-30% of the slide affected.
3- moderate	Thickening of alveolar walls by mixed inflammatory cells; up to 31-50% of the slide affected.

4- marked to severe	Thickening of alveolar walls by mixed inflammatory cells; >50% of the slide affected.
**Perivascular inflammatory infiltrates (cuffing)**	
0-normal	No significant findings.
1-minimal	Incomplete or loosely formed cuffs; rarely to occasionally observed.
2-mild	Numerous cuffs; predominantly incomplete and loosely formed with lesser well-formed cuffs.
3-moderate	Numerous cuffs; many ~ half being well-formed with broad dense cuffs.
4- marked to severe	Numerous cuffs; predominantly are well-formed with broad dense cuffs.
