# Supplementary material for: An Intranasal Challenge Model in African Green Monkeys (Chlorocebus aethiops) for Mild-to-Moderate COVID-19 Disease Caused by Subvariant XBB.1.5
Source: Viruses. 2025 Oct 14;17(10):1373. doi: 10.3390/v17101373 (PMC12568310; doi:10.3390/v17101373)
Supplement: Supplementary file 1 [file viruses-17-01373-s001.zip › Table S4 - Individual Animal Lung Ordinal Scores.pdf]

Table S4. Individual Animal Lung Ordinal Scores									
Subject		% lung area affected	Inflammation in bronchioles	Inflammation in alveolar spaces	Alveolar septal pneumocyte damage and repair, +/- hyaline membranes,	Alveolar interstitial inflammation	Perivascular inflammatory infiltrates	Cumulative score (each lobe)	Cumulative score (each [illegible]
01	Right cranial lung lobe	1	1	0	0	0	0	2	26
	Right caudal lung lobe	2	2	1	1	1	0	7	
	Right middle lung lobe	0	0	0	0	0	0	0	
	Right accessory lung lobe	2	2	1	0	1	2	8	
	Left cranial lung lobe	1	1	0	0	0	0	2	
	Left caudal lung lobe	2	2	1	0	1	1	7	
02	Right cranial lung lobe	1	1	1	0	0	1	4	30
	Right caudal lung lobe	1	1	0	0	0	0	2	
	Right middle lung lobe	1	1	1	0	0	0	3	
	Right accessory lung lobe	1	1	1	0	0	0	3	
	Left cranial lung lobe	2	2	2	1	1	1	9	
	Left caudal lung lobe	2	2	1	1	1	2	9	
03	Right cranial lung lobe	2	2	2	1	2	1	10	46
	Right caudal lung lobe	1	1	1	0	1	1	5	
	Right middle lung lobe	1	1	1	0	1	0	4	
	Right accessory lung lobe	1	1	1	0	1	1	5	
	Left cranial lung lobe	3	3	2	1	1	2	12	
	Left caudal lung lobe	2	2	2	1	1	2	10	
04	Right cranial lung lobe	3	3	3	3	2	2	16	98
	Right caudal lung lobe	4	4	3	3	3	2	19	
	Right middle lung lobe	4	4	3	2	2	3	18	
	Right accessory lung lobe	2	2	2	1	1	1	9	
	Left cranial lung lobe	4	4	3	4	3	2	20	
	Left caudal lung lobe	3	3	3	3	2	2	16	
